# Microslit on a chip: A simplified filter to capture circulating tumor cells enlarged with microbeads

**DOI:** 10.1371/journal.pone.0223193

**Published:** 2019-10-24

**Authors:** Seung Joon Lee, Tae Seok Sim, Hyun Young Shin, Jungmin Lee, Min Young Kim, Joseph Sunoo, Jeong-Gun Lee, Kyungmoo Yea, Young Zoon Kim, Danny van Noort, Soo Kyung Park, Woon-Hae Kim, Kyun Woo Park, Minseok S. Kim

**Affiliations:** 1 Department of New Biology, DGIST, Daegu, Republic of Korea; 2 CytoDx, Pangyo-ro, Beon-gil, Bundang-gu, Seongnam-si, Gyeonggi-do, Republic of Korea; 3 Samsung Electronics, Ltd., Maetan3-dong, Youngtong-gu, Suwon-si, Gyeonggi-do, Republic of Korea; 4 Division of Neurooncology and Department of Neurosurgery, Samsung Changwon Hospital, Sungkyunkwan University School of Medicine, Changwon, Republic of Korea; 5 Division of Biotechnology, IFM, Linköping University, Linköping, Sweden; 6 Daejeon Wellness Hospital, Beon-gil, Dongseo-daero, Daedeok-gu, Daejeon, Republic of Korea; 7 Translational Responsive Medicine Center, DGIST, Daegu, Republic of Korea; The Ohio State University, UNITED STATES

## Abstract

Microchips are widely used to separate circulating tumor cells (CTCs) from whole blood by virtues of sophisticated manipulation for microparticles. Here, we present a chip with an 8 μm high and 27.9 mm wide slit to capture cancer cells bound to 3 μm beads. Apart from a higher purity and recovery rate, the slit design allows for simplified fabrication, easy cell imaging, less clogging, lower chamber pressure and, therefore, higher throughput. The beads were conjugated with anti-epithelial cell adhesion molecules (anti-EpCAM) to selectively bind to breast cancer cells (MCF-7) used to spike the whole blood. The diameter of the cell-bead construct was in average 23.1 μm, making them separable from other cells in the blood. As a result, the cancer cells were separated from 5 mL of whole blood with a purity of 52.0% and a recovery rate of 91.1%, and also we confirmed that the device can be applicable to clinical samples of human breast cancer patients. The simple design with microslit, by eliminating any high-aspect ratio features, is expected to reduce possible defects on the chip and, therefore, more suitable for mass production without false separation outputs.

## Introduction

CTCs are generally seen as a prognostic indicator for patients with various metastatic carcinomas [1] and can act as a predictor of metastatic diseases [2], a disease which is more than 90% responsible for cancer related deaths [3]. As CTCs in blood are rare cell events (1–10 cells/mL of blood) [4], high recovery and purification rates are essential for clinical applications including diagnostics, prognostics, and monitoring tumor recurrence and therapeutic drug responses [5]. For example, the number of CTCs can be associated with the survival time of the patient after therapy [6]. Therefore, high recovery rate is essential to assure a better prediction. Also, CTCs can be potentially useful as markers in early diagnostics for a number of primary tumors, including lung, neuroendocrine, breast and pancreas [7–10]. As such, CTCs can be used as a diagnostic tool for personalized treatment [11]. However, currently, capturing extremely rare and heterogeneous CTC populations from patients’ blood samples is still challenging in their purity and recovery rate.

There are, currently, two basic methods to isolate CTCs; immun0affinity, typically by using the EpCAM antigen as a target molecule, and filtration-based technologies, which are based on the size differences between CTCs and other cells in whole blood [12]. While the first method displays high sample purity (>50%), it shows relatively low capture efficiency [13–15]. Contrary, the latter shows high capture efficiency (>90%), but poor sample purity [16–20]. Examples of both systems include: immunoaffinity-based devices making use of antibody coated magnetic beads [21–27]; silicon micropillars binding anti-EpCAM expressing CTCs [13]; size-dictated immunocapture chip with high performance [25]. Systems exploiting the physical properties of CTCs, such as density, size and deformability, include centrifugation [28–31], polycarbonate microfilters with 8 μm pore size [32, 33] and spiral microfluidics utilizing hydrodynamic forces [34, 35]. So far, the only system cleared by FDA, CellSearch^®^ (Menarini Silicon Biosystems Inc, PA, USA), has a relative low recovery rate of 80% [36].

**Fig 1:**
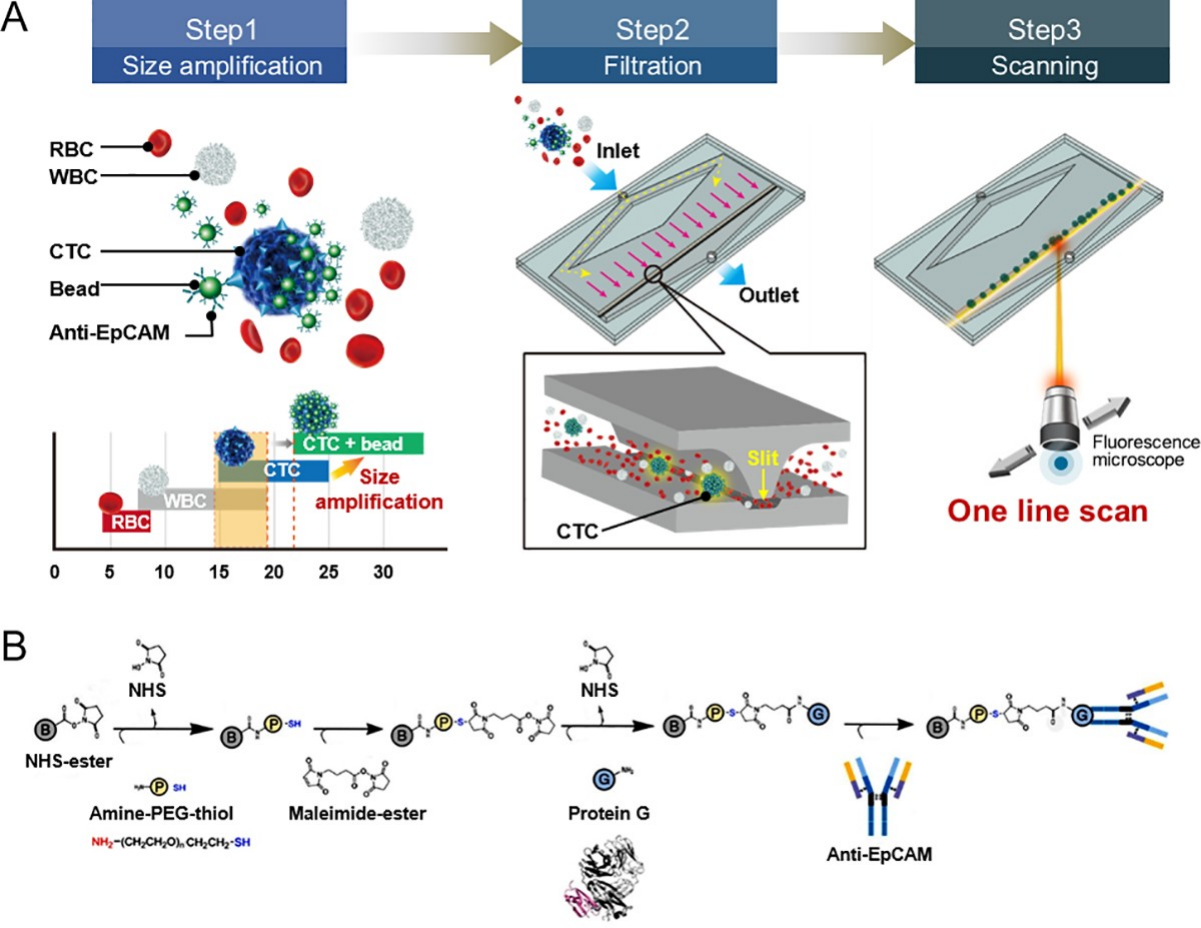
Schematic of the microslit filter chip to isolate circulating tumor cells. (**A**) The workflow of the microslit filter system consisting of 3 steps. Step 1 (size amplification): Cell size distribution in whole blood. WBCs and CTCs overlap. However, by conjugating the CTCs with functionalized microbeads, the WBCs and CTC-bead constructs are distinguishable. Step 2 (filtration): The CTC-bead constructs are captured by the 8 μm deep microslit, while the other cells in the whole blood pass the slit. Step 3 (Scanning): CTCs are simply and rapidly identified with 1D scan. (**B**) Coupling chemistry of the bead functionalization, after the standard EDC/NHS coupling to a carboxyl-bead (B). Further steps in the process included a thiol-PEG-amine (P), maleimide-ester, protein G (G) and finally, anti-EpCAM.

In our CTC separation strategy, the main objective was to develop a filter on a microfluidic chip lacking high-aspect ratio features, thus simplifying fabrication. As noted ahead, microfluidic filter chip technologies have exhibited high recovery rates, but they should comprise sophisticate filter gaps with high aspect ratio. These approaches are vulnerable to mass production and if the filters caused several parts of microstructure defects in a chip, it directly affects the performance of recovery rate. We developed a unique filter system consisting of one microslit with 27.9 mm width and 8 μm height to separate the CTC-bead constructs from whole blood, with the advantage of increased throughput and lowered stress on the captured CTCs. Due to the relative long width of the microslit, clogging has been minimized, which is one of the causes of low purity in other systems. Also, the microslit allows for faster cell identification, as the image simply can be acquired with 1-lane direction (1D) scanning (Fig 1A). We enhanced CTC separation by immobilizing 3 μm beads to MCF-7 cells (a model cell for the CTC), to overcome the size overlap of CTCs with white blood cells (WBCs). Finally, apart from microscopy, the separation procedure was fully automated to practically facilitate clinical utility. As a result of our separation technology, the cancer cells were separated from 5 mL of spiked whole blood with a purity of 52.0% and a recovery rate of 91.1%.

## Material and methods

### Fabrication of the microslit chip

We used 0.5 mm thickness, fine rounded and double side polished 6 inch BOROFLOAT 33 glass wafers (Schott AG Co., Germany) and the chip was fabricated from two glass wafers, used as top and bottom, as shown in S1 Fig. To remove small organic molecules, the two glass wafers were cleaned for 10 min in boiling Piranha solution (3:1 of H_2_SO_4_ and 30% H_2_O_2_), rinsed with DI water and dried with N2 gas. To remove residual water, the wafers were baked in an oven at 300°C for 20 min, after which 1000 Å amorphous silicon was deposited on the wafer, by low pressure chemical vapor deposition (LPCVD). Next, the amorphous silicon surface was patterned with photoresist (AZ P4330, Clariant Corp., NJ, USA) using standard photolithography and etched using reactive-ion etching (RIE). The exposed glass surface was etched in a 10% HF solution (0.85 μm/min), to a depth of 8 and 50 μm for the bottom and top wafer, respectively. The remaining resist and amorphous silicon were then stripped. Holes for the inlet and the outlet were sandblasted in the top wafer (S2 Fig). The depth and surface roughness of the etch glass were measured using a surface profilometer (KLA-Tencor, CA, USA). Finally, the top and bottom components were aligned and bonded at 600°C for 20 min in a furnace.

### Cell culture and bead preparation

In this study, MCF-7 breast cancer cell line (ATCC, VA, USA) was used as a model of CTCs in blood. The cell line was maintained in Dulbecco’s Modified Eagle Medium (DMEM) and RPMI-1640 supplemented with 10% fetal bovine serum, 100 IU/mL penicillin and 100 mg/mL streptomycin. The cells were cultivated in an incubator at 37°C and 5% CO2. Adherent cells were harvested by trypsinizing the culture before it reached confluency.

All reagents and microbeads were purchased from Sigma-Aldrich, MO, USA, unless otherwise stated. To make EpCAM-conjugated microbeads, 100 mg of carboxylate modified melamine 3 μm microbeads were washed twice with 0.1 M morpholinoethanesulfonic acid (MES) buffer (pH 5.5). 0.2 M EDC and 0.05 M NHS (Sigma-Aldrich) were added to the MES buffer containing the beads, after which the mixture was shaken for 30 min and centrifuged to remove unreacted materials. 1 mM heterobifunctional PEGs (thiol-PEG-amine, Creative PEGWorks, NC, USA) in 1 mL of PBS buffer (pH 7.4, Invitrogen) was then added to the beads, shaken for 1 hr and centrifuged to remove unbound PEGs. The thiol-functionalized microbeads were incubated with 1 mM of 4-Maleimidobutyric acid N-hydroxysuccinimide ester in PBS for 20 min at room temperature. Next, the beads were mixed with 50 μg/mL of protein G in PBS for 2 hrs at room temperature, followed by rinsing with deionized water. 1 mg/mL of anti-EpCAM antibodies (R&D Systems, MA, USA) in PBS buffer was then added to the microbead aliquot and incubated at 4°C overnight. Finally, the antibody-conjugated microbeads were washed in PBS buffer to remove any non-specifically bound antibody and the remaining binding sites were blocked with 2% BSA solution (Fig 1B).

### Blood preparation

**Fig 2:**
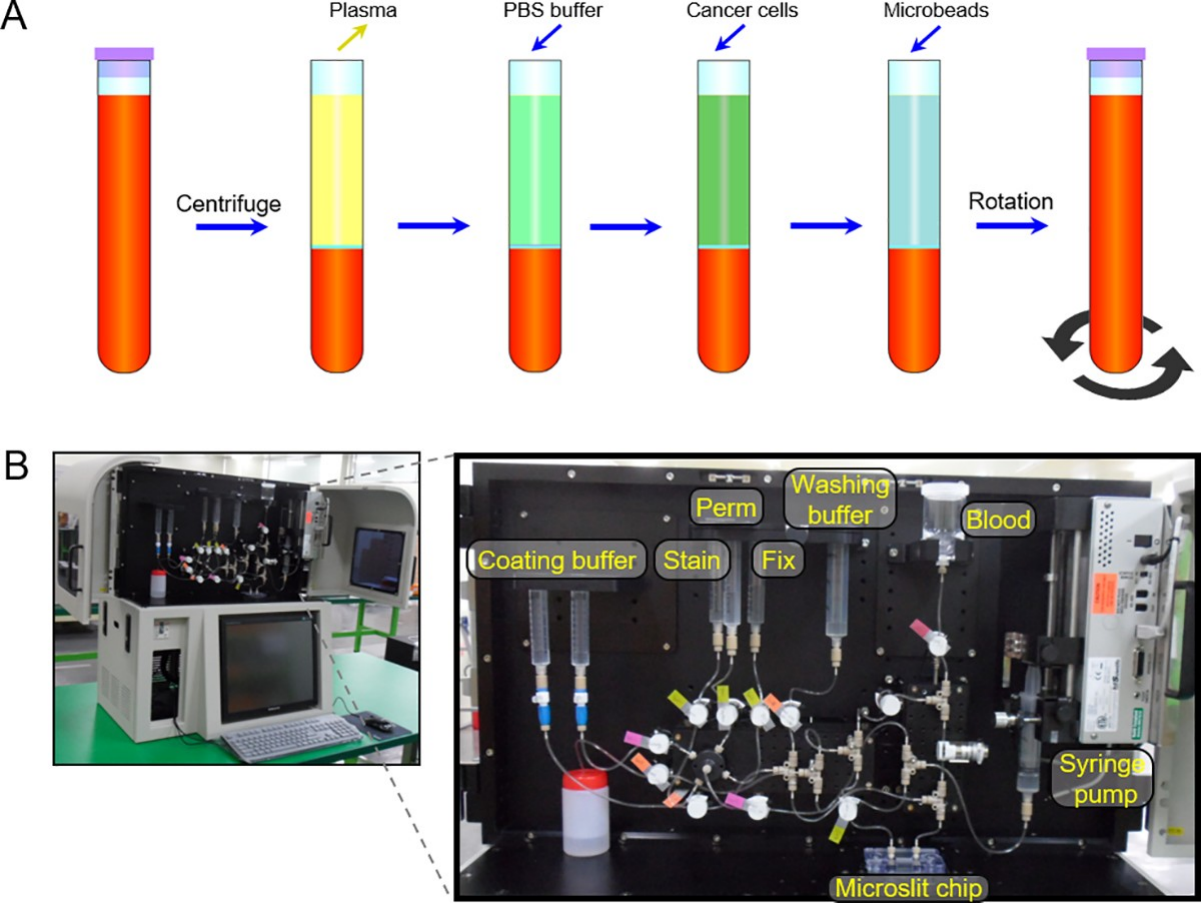
Sample preparation process and the automated CTC isolation system. (**A**) Blood sample preparation. Whole blood is centrifuged after which the blood plasma is removed. PBS buffer is added and then cancer cells are added. To facilitate the capture of the cancer cells in the chip, microbeads are added. Finally, the blood and additions are mixed by rotation. (**B**) Automatic set-up. All tubes connected to individual reservoirs were opened and closed with solenoid valves, while the blood reservoir also includes a shaker. The preprocessed sample was inserted to the blood reservoir and then microfluidic isolation and staining processes were conducted by just one click.

Human blood samples were obtained and approved by the Institutional Review Board (IRB) at Yonsei University and Daejeon Wellness Hospital in Korea. In all cases, informed written consent was obtained from the participants. The plasma of 5 mL whole blood was removed by centrifuge and aspiration. A buffer of PBS and 2 mM EDTA was added to complement the volume of the removed plasma. 100 MCF-7 cancer cells were used to spike the blood sample after which it was mixed. Next, 60 μL (6 × 10^9^ beads per mL) of the microbead solution was added to the blood sample and incubated on a rotator at 12 rpm for 1 hr at room temperature. Finally, the 5 mL blood sample was transferred to the blood reservoir in the automated liquid handling system (Fig 2A).

### Flow simulations

The flows were simulated using COMSOL multiphysics (Comsol Inc., MA, USA). The fluid’s viscosity was the same as blood (5 cP) [37]. The effect of the pressure caused by CTC-bead constructs captured in the slit was also simulated. The CTC-bead constructs were 25 μm in diameter, while the flow rates ranged from 50 μL/min to 1 mL/min. The deformability of the cells was not considered in this simulation. The model for slit and filter separation, had a chamber size of 540×1200×50 μm (w×l×h). The slit had a gap of 8 μm height and 5 μm deep, while the filter consisted of 8×50×50 μm (w×l×h) gaps spaced 100 μm apart. The pressure was simulated by assuming a uniform flow rate over the width of the chamber.

### Automation

The CTC separation and cell staining procedure were automated as shown in Fig 2B. Solenoid valves controlled all the reservoirs connected to the input of the microslit chip, while a syringe pump was connected to the outlet of the chip. By operating the pump with withdrawal mode, a negative pressure was created, ensuring flows from the reservoirs, through the chip to the outlet. To prevent blood coagulation and sedimentation of the blood cells, including the CTCs, the blood reservoir was attached to a stepping motor performing a swinging motion.

### Cell separation, staining and analysis

The pretreated blood was loaded into the liquid handling system, flowing through the microslit chip at 100 μL/min for 50 min, followed by a PBS wash. The captured cells were then fixed by flowing 1% paraformaldehyde (PFA) in PBS at 50 μL/min for 20 min and then, for permeabilization, 0.01% Triton X-100 reagent at 50 μL/min for 10 min. A mixture of 4',6-diamidino-2-phenylindole (DAPI), anti-cytokeratin PE (CK; CAM 5.2, BD Biosciences, CA, USA) and CD45 FITC (BD Biosciences, CA, USA) was used for staining and flowed into the chip at 15 μL/min for 30 min, followed by PBS wash. The stained cells were imaged by inverted microscope (Olympus IX81-ZDC, Japan). CD45 negative, DAPI and CK positive cells were counted as CTCs, while CK negative, DAPI and CD45 positive cells were counted as WBCs.

To measure the diameters of the breast cancer cells and the cancer cells bound to microbeads, we used the inverted microscope. The breast cancer cells were inserted in a 12 well plate and the plate were gently centrifuged (1000 rpm for 3 min). When the cells reached to the bottom plate, images were randomly taken with 20× magnification and the diameter of the cancer cells was measured with the MetaMorph image software (MetaMorph Inc., TN, USA). The average and standard deviation values were calculated from the 50 cell diameter values. The microbead-conjugated cancer cells were also measured in the same manner as the procedure used with the original cancer cells.

## Results and discussion

### Chip and glass surface roughness

**Fig 3:**
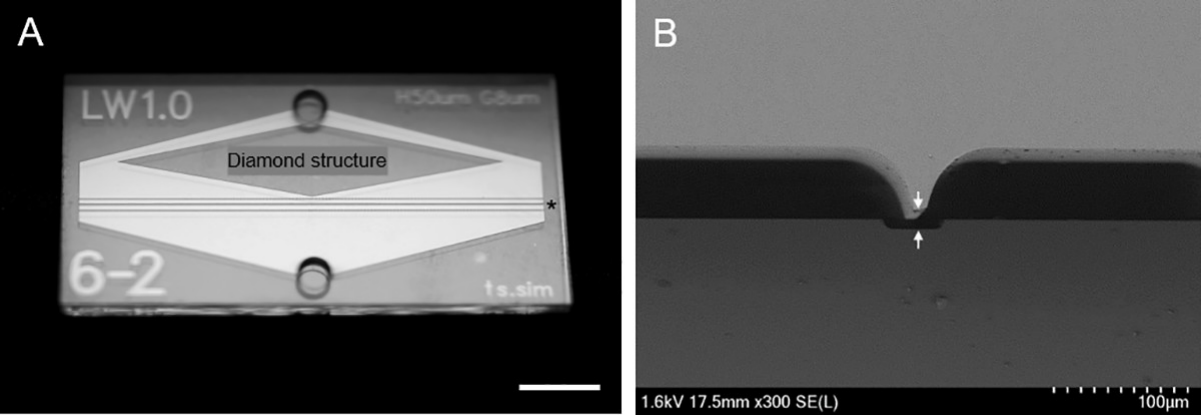
Fabricated microslit filter chip. (**A**) An image of the microslit filter chip. The asterisk (*) indicates microslit filter area and a diamond shape structure is located to make uniform flow distribution between the inlet port and slit area, respectively. Scale bar, 5 mm. (**B**) A cross-section view: scanning electron microscope image of the microslit device (scale bar: 100 μm). The white arrows indicate the depth of microslit and the value was 8.10 μm in average.

The microslit chip could be fabricated as following the above process (Fig 3A). In many microfluidic applications, polydimethylsiloxane (PDMS) has been widely utilized by its benefits including easy, fast and cost-effective fabrication process as well as relevance of biological studies via biocompatibility, transparency, low autofluorescence and semipermeability. This is no exception in the field of CTC isolation [25, 34–36]. PDMS microchips, however, have critical drawbacks particularly in terms of commercial products: (1) PDMS shrinks during curing, (2) the cured PDMS is readily swelled, (3) mechanical and physical properties changes over time, (4) the softness limits aspect ratio of microstructures, increasing the probability of microstructure defects, (5) the hydrophobic surface requires very careful handling for fluid flows and absorbs various proteins and small hydrophobic molecules. To avoid the limitations of PDMS, we fabricated glass microchips to increase technical completeness for mass production. To establish the uniformity of the microslit, the variation of the depth of the slit was measured by using a surface profilometer. The roughness of the bottom glass surface was measured over a 4.0 × 4.0 μm^2^ area. The average depth of the slit was 8.10 μm (see the white arrows of Fig 3B), with a standard deviation of 0.12 μm (*n* = 50). The average surface roughness of the etched glass wafer, represented by the arithmetic mean value Ra [38], was less than 0.1 μm, while that of untreated glass was around 0.02 μm. This means that variation in roughness is less than the uniformity of the slit depth and does not impact the depth of the 27.9 mm wide slit, nor its capture performance (S2 Fig).

### Optimization of the microslit chip

**Fig 4:**
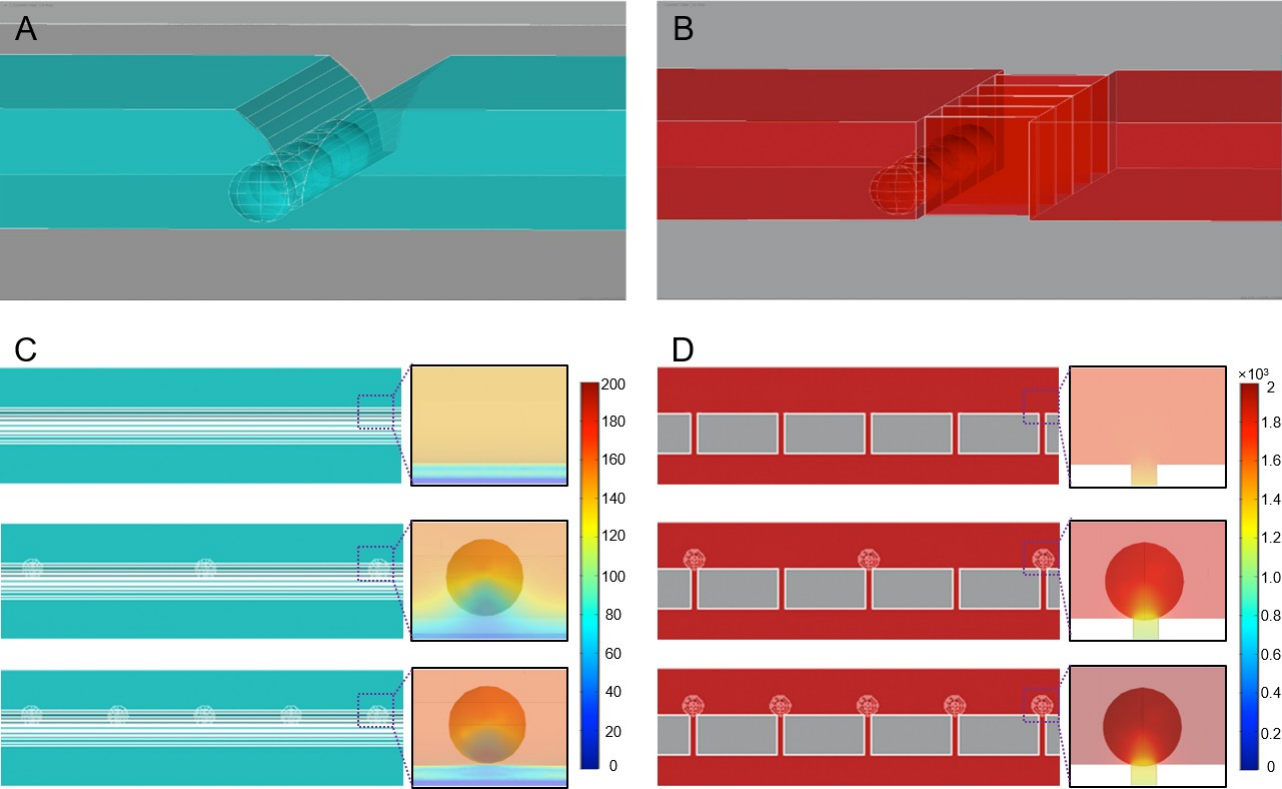
Simulation of two different separation models, with a side and top view, including 5 CTCs with 25 μm diameter. (**A**) The green model has a slit with a 8 μm height, a 5 μm width and 27.9 mm length. (**B**) The red model has a filter consisting of an 8 μm gap, a 50 μm height at 100 μm distance from each other. The filter itself is 27.9 mm long. (**C**) Top view of the green slit-filter model showing the positions of the 0, 3 and 5 CTCs. The pressure profiles around the CTC are exhibited. The pressure ranges from 140, 152 to 161 Pa for 0, 3 and 5 cells, respectively. (**D**) Top view of the red filter model showing the positions of the 0, 3 and 5 CTCs. The pressure distributions on the CTC surfaces are shown. The pressure range from 1652, 1820 and 1955 Pa for 0, 3 and 5 cells, respectively.

One of the advantages that a microslit approach has, instead of a microfilter with gaps [14, 23], is it reduces pressure built up with increase of the captured CTCs, when compared to other systems utilizing 8 μm gaps or pores (Fig 4). There will be less pressure exerted on the captured CTCs, which is important for cell viability when culturing the separated CTCs for further analysis. Also, there is less chance that a CTC will slip through the filter. Simulations showed that the fluidic pressure was almost 10 times less exerted in the microslit approach (Fig 4A) when comparing to a filter with 8 μm gaps spaced 100 μm apart (Fig 4B), which was modeled with similar geometry condition of the previous filter studies [23, 24]. In addition, as the number of captured cells increases, the tendency was not changed and more pressure increase on the cell membrane was exhibited in the filter type (Fig 4C and 4D). To have a comparable pressure in the filter design as in the slit design, the flow rate should be lowered. This affects the throughput of the system. The slit model, therefore, is able to perform under a higher throughput, with less stress on the captured CTCs. In addition, as the pressure is inversely proportional to the third power of the channel width, the reduction of flow path by capturing CTC-bead constructs can lead to a significant increase of pressure. When 100 CTC-bead constructs are captured, the microslit was only 8.3% blocked. However, the pressure increase was 33% in 1 mL/min flow rate (S3 Fig). A previously published CTC microfilter device reported a pressure of 3.45 kPa while the cell membrane tension was estimated to be 13.8 mN/m [16]. Since the critical pressure differs in cell types [39], we found that 100 μL/min flow rate was the condition not to make captured CTCs harmful under isolation process.

In addition, uniform fluidic velocity should be distributed to the filter area to secure the reproducibility of microfluidic filter because the capture of deformable particles such as cells is the combinational result of fluidic force, filter gap and cell sizes. However, few studies have considered the uniformity of flow velocity in CTC isolation technologies. The chip was specially designed to have a uniform flow along the length of the microslit based on our previous work [40]. To accomplish this, a diamond shape barrier was placed between the input and the slit (S1 Movie).

### Isolation performance of the microslit chip

To avoid size overlap between CTCs and blood cells [approximately for RBCs (6.2 ~ 8.2 μm in diameter) [41] and WBCs (7 ~ 20 μm in diameter)] [42, 43], we used EpCAM-conjugated microbeads with 3 μm in diameter. By the size amplification process, the breast cancer cells increased in diameter from 16.5 ± 1.9 μm to a substantially enlarged 23.1 ± 2.6 μm (*n* = 50) which is above the upper limit for WBC size. With this microbead conjugation technique, therefore, we could avoid size overlap between these two types of cells. These constructs gave rise to not only more clear size discrimination between CTCs and leukocytes but also larger stiffness of CTCs, making for CTCs more difficult to pass through the slit.

**Fig 5:**
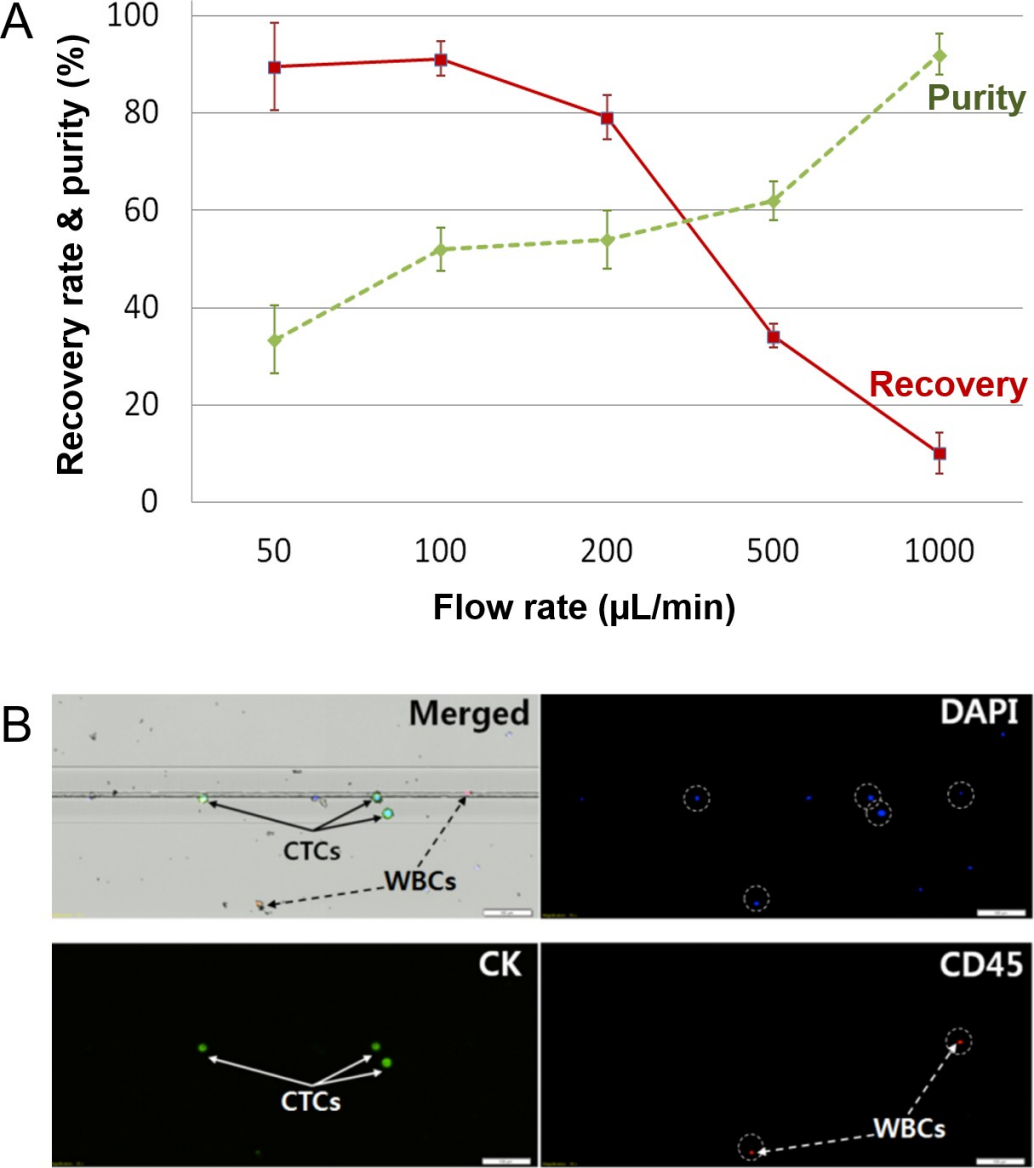
Performance of the automated microslit filter system. (**A**) Optimization of recovery rate and purity. Higher flow rates lead to higher purity by lower recovery rate. (**B**) Images of the captured WBCs and cell-bead constructs. By staining the cells, CTCs and WBCs can be identified. CTC stains positive for CK and DAPI, but negative for CD45. WBCs represent DAPI (blue) and CD45 (red) double positive cells (Scale bar: 100 μm).

Using 5 mL of whole blood, we measured the number of WBCs at different flow rates. At flow rates less than 50 μL/min, there were thousands of WBCs, while at flow rates over 500 μL/min, there were only a few WBCs in the recovered sample. At low flow rates, there will not be enough force to push the larger WBCs through the microslit, while the WBCs tend to stick to the surface of the channels. As expected, recovery rate exhibited inverse tendency with purity. As shown in Fig 5A, the purity gradually increased with faster flow rates and reached to almost 100% while the recovery rate declined at faster flow rates, after peaking at 100 μL/min. Based on the results, we chose 100 μL/min flow rate as the optimal condition in this system, whose condition also satisfies intact CTC isolation according to the above simulation. In this condition, the system showed excellent performance with over 91.1% recovery rate and 52% purity. It is noted that this achievement is meaningful in aspect of commercialization because the data were acquired from the automated system and the microslit is much favorable to mass production rather than a microfilter composed of many microstructures to make micro gaps, reducing defect rates among products. In addition, as only one-line imaging is required (Fig 5B) after simple detachment of microslit chip from a cartridge (Supporting Information S4 Fig), the microslit approach could have much faster identification for cell enumeration analysis while conventional microfilter methods had to be taken for very wide range of microfilter area and required for big data storage.

**Fig 6:**
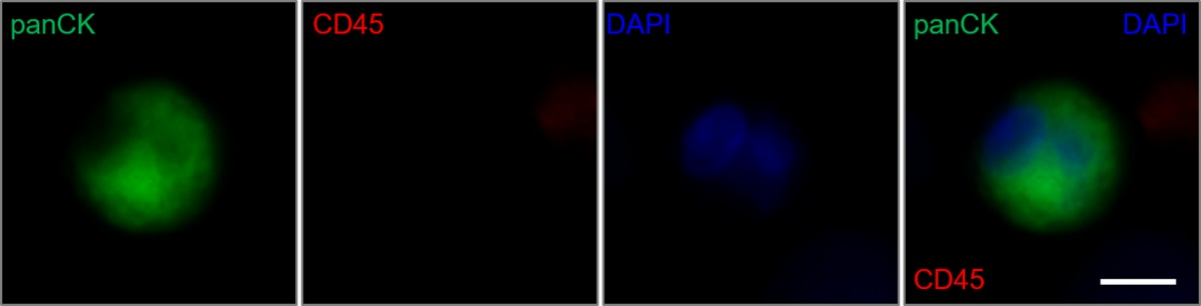
Immunocytochemistry images to evaluate a CTC from whole blood of an advanced breast cancer patient. A captured CTC on the microslit was immunostained with pan-cytokeratin (panCK, green), CD45 (red) and DAPI (blue). CTCs were detected with panCK (+), CD45 (-) and DAPI (+) and a representative sample was shown (Scale bar, 10 μm).

Since the hematocrit is different for each person, we compared recovery rates of buffer and whole blood to see the effect of blood cells in terms of recovery rate. Table 1 shows the results of the purity and recovery rate of the captured cancer cells. After 5 repeats, the results presented there was a slightly lower capture rate of the cancer cells in whole blood compared to buffer, 91.1% as compared to 95.6%, respectively. The reduction of 4% recovery rate could be predicted that captured CTCs might be escaped by pushing back the blood cells from behind. However, the result for comparison between the extreme cases was not significantly different, seeming to be negligible for the individual. To validate whether the microslit device could be applied to clinical samples, we acquired peripheral blood from an advanced and metastatic stage IV breast cancer patient. After bead binding process with 2mL of patient blood, the sample was injected into the microslit device. After washing the microfluidic channel with PBS, immunofluorescence imaging was performed. As shown in Fig 6, we could observe a CTC in the microslit structure, implying that the platform has a potential to be utilized as a clinical application.

**Table 1:** Comparison of recovery rate and purity with respect to the sample. Cancer cells were spiked into the whole blood sample to target the injection of 100 cells after which it was mixed (*n* = 5; SD is the standard deviation; CV is the coefficient of variation).

Sample	Input	Captured	SD	CV (%)	Recovery rate (%)	Purity (%)
Buffer	103.5	99.0	13.0	13.1	95.6	-
Whole blood	110.2	100.4	5.7	5.8	91.1	52.0

In this study, we used MCF-7 cells as a model of the CTCs. CTCs have been known as highly heterogeneous populations in terms of marker expressions, cells sizes and genetic mutations [5]. Although this study has a limitation, which has not been fully covered the heterogeneity of CTCs by using one type of cancer cells, other research works [23, 44–46] have used the cell line to evaluate the performance of separation systems. As a further study, however, much effort will be required for more close mimic of real CTC samples by implementing various types of cancer cells and multiple antibodies to capture EpCAM negative targets.

## Conclusions

In this paper, we presented a novel CTC separation method showing both high purity and high recovery rate. This was achieved by combining affinity binding of microbeads to the cancer cells and a microslit filtration system. Due to the increased size of the cell-bead constructs they were clearly separable from the WBCs.

Furthermore, because of the ultra-wide width of the microslit, blood cells could easily bypass the captured cancer cell-bead constructs, as there was limited clogging of the slit. Also, the automated system with a microslit allows for higher throughput and lower cellular stress than a system with a filter. As such we were able to attain a purity of 52% and a recovery rate of 91.1% for the cancer cells. It was, however, possible to attain a higher purity with our system by increasing the flow rate, but that would result in a lower recovery rate. In fact, that makes our system tunable to the task required.

Finally, the simplicity of the slit design, by eliminating any high-aspect ratio features, reduces possible defects on the chip and, therefore, false separation outputs. Especially when thinking about large volume production, this could reduce production costs and increase the reliability of the system.

## Supporting information

S1 Fig Fabrication of the microslit filter chip. (TIF)

S2 Fig Images of the inlet and the outlet for the fabricated microslit device. (TIF)

S3 Fig The simulation result of the inlet pressure as function of the flow rate in the microslit device. Higher flow rate leads to higher pressure in the microfluidic chamber.
(TIF)

S4 Fig (A) An assembled image with a microslit device and the cartridge (B) An image after detaching the cartridge. The microslit device was simply separated.
(TIF)

S1 Movie The process of whole blood injection into the microslit chip. The cancer cells were captured in the microslit area.
(MP4)
