# Supplementary figures and images for: Microslit on a chip: A simplified filter to capture circulating tumor cells enlarged with microbeads
Source: PLoS One. 2019 Oct 24;14(10):e0223193. doi: 10.1371/journal.pone.0223193 (PMC6812780; doi:10.1371/journal.pone.0223193)

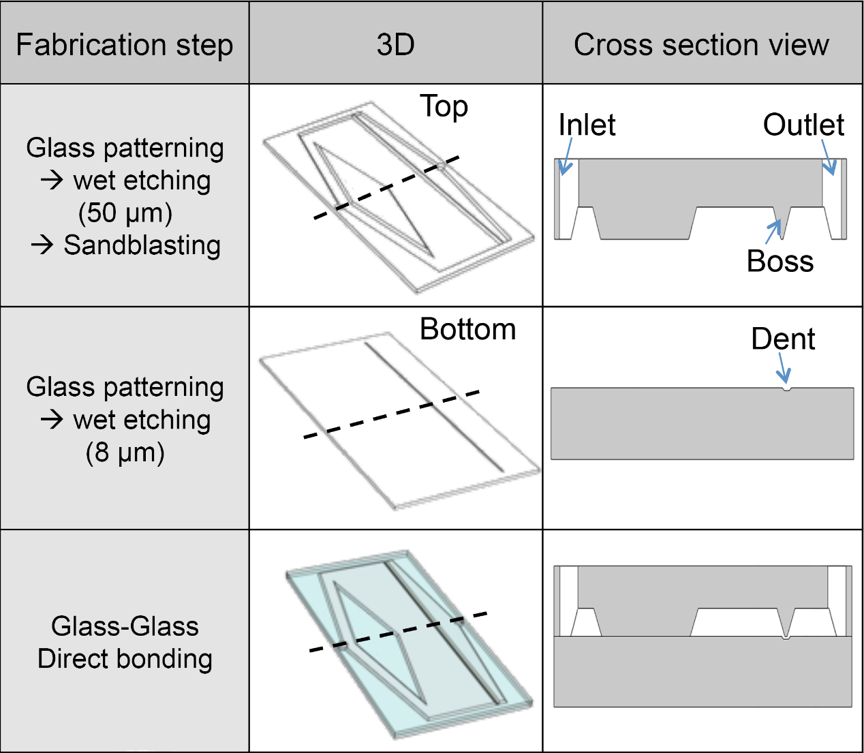

Supplement: S1 Fig — (TIF) [file pone.0223193.s001.tif]

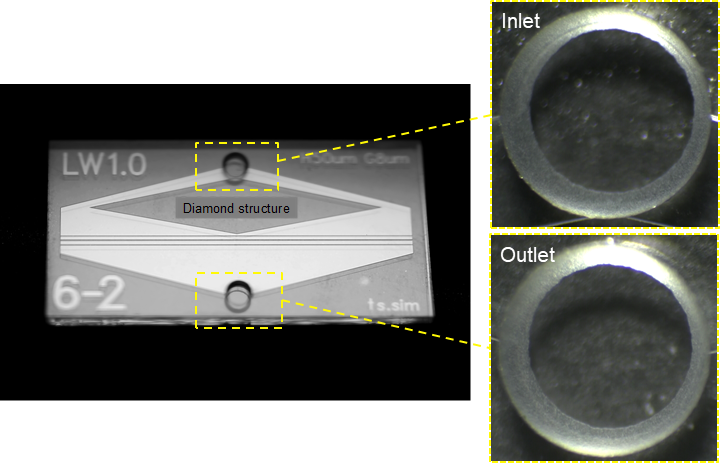

Supplement: S2 Fig — (TIF) [file pone.0223193.s002.tif]

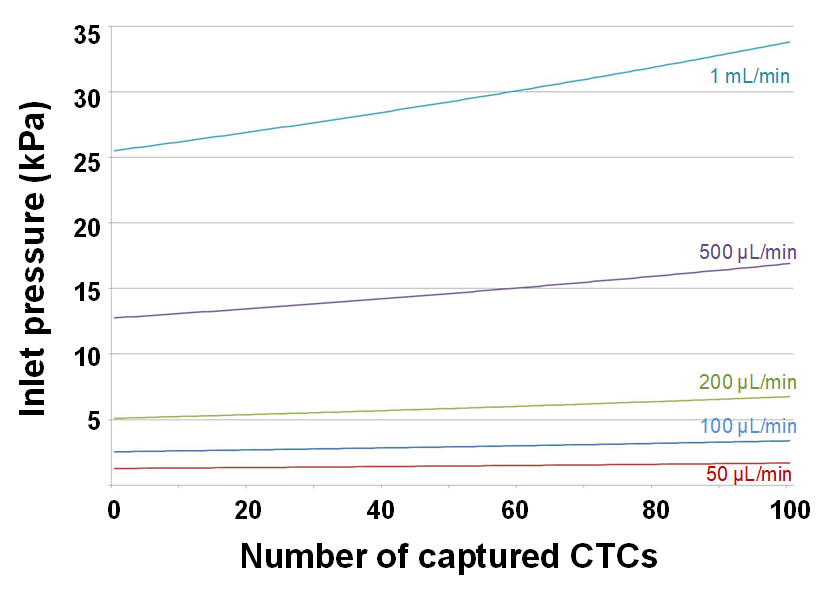

Supplement: S3 Fig — Higher flow rate leads to higher pressure in the microfluidic chamber. (TIF) [file pone.0223193.s003.tif]

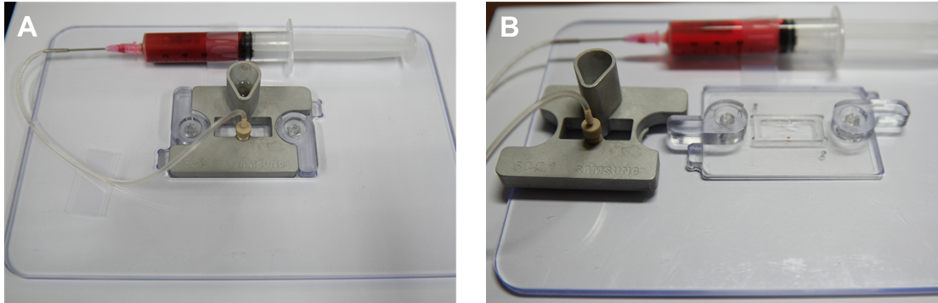

Supplement: S4 Fig — (A) An assembled image with a microslit device and the cartridge (B) An image after detaching the cartridge. The microslit device was simply separated. (TIF) [file pone.0223193.s004.tif]
